# Association of acidic urine pH with impaired renal function in primary gout patients: a Chinese population-based cross-sectional study

**DOI:** 10.1186/s13075-022-02725-w

**Published:** 2022-01-25

**Authors:** Yuwei He, Xiaomei Xue, Robert Terkeltaub, Nicola Dalbeth, Tony R. Merriman, David B. Mount, Zhe Feng, Xinde Li, Lingling Cui, Zhen Liu, Yan Xu, Ying Chen, Hailong Li, Aichang Ji, Xiaopeng Ji, Xuefeng Wang, Jie Lu, Changgui Li

**Affiliations:** 1 Shandong Provincial Key Laboratory of Metabolic Diseases and Qingdao Key Laboratory of Gout, The Affiliated Hospital of Qingdao University, Qingdao, 266003 China; 2 Department of Endocrinology and Metabolism, The Affiliated Hospital of Qingdao University, Qingdao, China; 3 Shandong Provincial Clinical Research Center for Immune Diseases and Gout, Qingdao, China; 4 Institute of Metabolic Diseases, Qingdao University, Qingdao, China; 5 VA San Diego Medical Center, San Diego, USA; 6 Department of Medicine, University of California San Diego, La Jolla, CA USA; 7 Department of Medicine, University of Auckland, Auckland, New Zealand; 8 Division of Clinical Immunology and Rheumatology, University of Alabama Birmingham, Birmingham, Alabama USA; 9 Department of Biochemistry, University of Otago, Dunedin, New Zealand; 10 Renal Divisions, Brigham and Women’s Hospital and VA Boston Healthcare System, Harvard Medical School, Boston, Massachusetts USA; 11 Department of Nephrology, Chinese PLA General Hospital, Chinese PLA Institute of Nephrology, State Key Laboratory of Kidney Diseases, National Clinical Research Center for Kidney Diseases, Beijing, China; 12 Department of Nephrology, The Affiliated Hospital of Qingdao University, Qingdao, China

**Keywords:** Primary gout, Urine pH, eGFR, Nephrolithiasis, Microhematuria

## Abstract

**Background:**

Patients with gout frequently have low urinary pH, which is associated with the nephrolithiasis. However, the specific distribution of urinary pH and potential relationship of acidic urine pH to broader manifestations of kidney disease in gout are still poorly understood.

**Methods:**

A 2016–2020 population-based cross-sectional study was conducted among 3565 gout patients in the dedicated gout clinic of the Affiliated Hospital of Qingdao University to investigate the association between low urinary pH and kidney disease. We studied patients that we defined to have “primary gout”, based on the absence of > stage 2 CKD. All subjects underwent 14 days of medication washout and 3-day standardized metabolic diet. We obtained general medical information, blood and urine biochemistries, and renal ultrasound examination on the day of the visit. The primary readouts were urine pH, eGFR, nephrolithiasis, renal cysts, microhematuria, and proteinuria. Patients were assigned into 5 subgroups (urine pH ≤5.0, 5.0 <pH≤ 5.5, 5.5 <pH< 6.2, 6.2 ≤pH≤ 6.9, and pH >6.9), aligning with the clinical significance of urine pH.

**Results:**

Overall, the median urine pH and eGFR of all patients was 5.63 (IQR 5.37~6.09), and 98.32 (IQR 86.03~110.6), with acidic urine in 46.5% of patients. The prevalence of nephrolithiasis, microhematuria, and proteinuria were 16.9%, 49.5%, and 6.9%, respectively. By univariate analysis, eGFR was significantly associated with age, sex, duration of gout, tophus, body mass index, systolic blood pressure, diastolic blood pressure, fasting blood glucose, total cholesterol, serum utare, hypertension, diabetes, and urine pH. On multivariable analysis, eGFR was associated with age, sex, diastolic blood pressure, serum uric acid, hypertension, diabetes, and urine pH. Acidic urine pH, especially urine pH < 5.0, was significantly associated with the prevalence of kidney disease, including > stage 1 CKD, nephrolithiasis, kidney cyst, and microhematuria. Patients with 6.2 ≤ urine pH ≤ 6.9 and SU ≤ 480 μmol/L had the highest eGFR with the lowest prevalence of nephrolithiasis, microhematuria, and proteinuria.

**Conclusions:**

Approximately half of gout subjects had acidic urine pH. Urine pH < 5.0 was associated with significantly increased nephrolithiasis, renal cyst, microhematuria, and proteinuria. The results support prospective clinical investigation of urinary alkalinization in selected gout patients with acidic urine pH.

**Supplementary Information:**

The online version contains supplementary material available at 10.1186/s13075-022-02725-w.

## Background

Gout is a highly prevalent disorder [1] that is commonly associated with uric acid and calcium oxalate nephrolithiasis [2], chronic kidney disease (CKD), and elevated risk of CKD progression [3] that appears accelerated in those palpable tophaceous disease [4]. Additional manifestations of kidney disease in gout include intermittent proteinuria, microscopic microhematuria, impaired renal concentrating function, and an increase in nocturia [5]. In addition, non-ionic forms of urate can promote uric acid and oxalate nephrolithiasis, which are in turn associated with CKD [6, 7]. In gout patients, associated hypertension and type 2 diabetes; hyperuricemia-mediated renal damage; tubular precipitation of urate microcrystals with—in the most severe forms of undertreated tophaceous gout—monosodium urate crystal deposition in the medulla; and toxic effects of analgesics used to treat gout flares [8, 9] are among the mechanisms that could contribute to CKD progression.

Urine pH normally is slightly acidic (averaging ~6.0), and acidic urine has been defined as pH below 5.5 [10]. Acidic urine is multifactorial in etiology; in gout patients, urine pH would be anticipated to be significantly influenced by factors including diet, insulin resistance, hydration status, and medications including thiazide diuretics and CKD [11–14]. Gout patients excrete urine with a significantly lower pH [15]. The urine pH has a direct effect on renal tubular lumen uric acid crystal formation [16]. Acidic urine pH also has been implicated in renal tubulointerstitial damage and proteinuria [17]. Moreover, acidic urine pH has been reported to be associated with a higher all-cause mortality (hazard ratio 2.550, 95% confidence interval (95% CI) 1.316–4.935) [10]. However, prior published studies have not systematically investigated the distribution and associations of urine pH in gout patients, or the associations in gout patients of renal function with differing urine pH. Moreover, clinical management guidelines in gout vary widely in recommendations for therapeutic attention to urine pH. Specifically, some guidelines have advocated that gout patients, especially those using uricosuric agents and recurrent stone formers, should receive therapy to alkalinize urine [18, 19]. In contrast, due to lack of hard clinical research evidence, the 2020 American College of Rheumatology (ACR) guidelines for management of gout did not recommend alkalization of urine pH in patients with gout [20]. Given the gaps in clinical evidence in this area, the objectives of this cross-sectional study were to identify the distribution of urine pH values in gout patients and to test the hypothesis that acidic urine pH is an independent risk factor for renal alterations in gout patients.

## Patients and methods

### Study participants and design

We conducted a population-based cross-sectional study of patients in the dedicated gout clinic of the Affiliated Hospital of Qingdao University during 2016–2020. The study was conducted according to the principles from the Declaration of Helsinki and was approved by the Ethics Committee of the Affiliated Hospital of Qingdao University. All patients who were involved in this study provided written informed consent. We enrolled 11,757 patients who met the 2015 ACR/European League Against Rheumatism (EULAR) classification criteria for gout [21]. Subjects were excluded for the following reasons: (i) acute phrase of gout flare (*n* = 3015), (ii) severe gout with inability or secondary gout (*n* = 3040), and (iii) unable to tolerate 14 days washout of medications (*n* = 1589). Those excluded because of contraindication to 14 days of medication washout included subjects with diabetes, hypertension, and > stage 2 CKD. We also excluded those who underwent gout flares frequently during washout (*n* = 56) were not able to follow the fixed metabolic diet (*n* = 84), withdrew consent (*n* = 217), had a nonstandard 14-day medication washout (*n* = 76), had incomplete urinary test data (*n* = 71), or were missing urine pH values (*n* = 44). Ultimately, 3565 primary gout patients were included. Patients were assigned into 5 subgroups (acidic urine pH ≤ 5.0, decreased urine pH of 5.0 < pH ≤ 5.5, 5.5 < pH < 6.2, 6.2 ≤ pH ≤ 6.9, and pH >6.9), aligning with the clinical significance of urine pH. 6.2 ≤ pH ≤ 6.9 as the treatment target of urine alkalization [22], with urine pH 5.5 or lower defined as “acid urine” [10].

Patients enrolled were required to conduct a 24-h urine collection after a 2-week washout. During the 2-week washout period, all enrolled patients were required to stop taking all drugs for 2 weeks after clinicians assessed that they tolerated medicine withdrawal. Patients were required to maintain a fixed metabolic diet during the last 3 days of the washout period. The diet included most sugars, starches, and fats. Protein is mainly provided by defatted or low-fat milk, eggs, and cereals. Protein and lipid intakes were 50~70 g/day and <50 g/day, respectively. The purine intake was less than 200 mg/day. The recommended fluid intake is more than 2000 mL/day. Total daily calorie intake and calorie allocation per meal were calculated based on height, weight, and work intensity of subjects (Supplementary Table 1). For the first morning urine, patients were instructed to collect their 10-mL clean mid-catch urine.

### Variables measured

General information including age, height, weight, smoking history (≥ 20 packs of cigarettes in a lifetime or ≥ one cigarette per day for ≥ one year), and alcohol history (alcohol intake ≥ once a week for 6 months). Blood pressure were measured using an Omron electronic sphygmomanometer (Omron, HBP-1300). Serum alanine aminotransferase (ALT), serum aspartate aminotransferase (AST), serum fasting blood glucose (FBG), serum triglyceride (TG), serum cholesterol (TC), high-density lipoprotein (HDL), low-density lipoprotein (LDL), serum urate (SU), serum creatinine (sCr), 24-h urine volume (recorded by patients), 24-h urine uric acid (uUA), and urine creatinine (uCr) were measured using an automatic biochemical analyzer (TBA-40FR, Toshiba Company, Japan). Proteinuria was measured using a urine automatic analyzer (AX-4280, ARKRAY Company, Japan), proteinuria positive referred to the concentration exceeds 0.15 g/day. Microhematuria was define as >3 red blood cells per high-power field on microscopic evaluation of a single, properly collected urine specimen [23]. Urine pH was directly measured using a pre-calibrated pH electrode (FE28-STANDARD, METTLER Toledo Company, Zurich, Switzerland).

A renal tract ultrasound examination using a standard imaging protocol was done on the day of the visit. The Philips IU22 color ultrasound instrument with a L12-5 broadband probe was used. The size, surface smoothness, parenchyma (cortical and medulla) echoes of both kidneys, and separate dilatation of both renal sinuses were observed in a single blind examination conducted by a senior ultrasound diagnostic physician. Renal cysts were defined as round anechoic areas with thin and smooth cyst walls and enhanced posterior echogenicity. Renal stones showed enhanced echogenicity. The presence of stone-free, single, or multiple kidney stones were detected by ultrasonography. Multiple kidney stones are defined as more than one stones in the kidney [24].

Body mass index (BMI) was calculated as weight in kilograms divided by height in meters squared. Kidney function measured by estimated glomerular filtration rate (eGFR; mL/min/1.73m^2^) and eGFR were calculated by CKD-EPI creatinine equation. GFR = 144 × (Scr/0.7)^-0.329^ × (0.993)^Age^, for female’s Scr < 62 μmol/L; GFR = 144 × (Scr/0.7)^-1.209^ × (0.993)^Age^, for female’s Scr > 62 μmol/L; GFR = 141 × (Scr/0.9)^-0.411^ × (0.993)^Age^, for male’s Scr < 80 μmol/L; and GFR = 141 × (Scr/0.9)^-1.209^ × (0.993)^Age^, for male’s Scr > 80 μmol/ L[25]. The fractional excretion of urate (FEUA) and 24-h urinary urate excretion (UUE) were calculated by 24-h urine volume and 24-h uUA and uCr. FEUA= uUA/uCr × sCr/sUA × 100% and UUE = uUA × 24-h urinary volume/(0.0061 × height (cm) + 0.0128 × weight (kg) – 0.1529) × 1.73 (mg/d/1.73m^2^)) [26]. The underexcretion type of hyperuricemia was defined as the FEUA <5.5% and UUE ≤600 mg */*day/1.73m^2^ [27].

### Statistical analyses

Data were presented as mean (standard deviation, SD) for continuous variables if normally distributed and median (interquartile range, IQR) if not normally distributed. For categorical variables, data were shown as number (percentage, %). For the comparison of general data of patients with different urine pH, ANOVA and chi-squared tests were used for continuous and categorical variables and the Kruskal-Wallis test was used for variables with a skewed distribution. Comparison of proportion in patients with different urine pH values, chi-squared tests were used. The chi-square test of 2×2 used for pairwise comparison between groups and significance was corrected by Bonferroni.

Univariate linear regression analyses were conducted to evaluate the respective relation between patients’ characteristics and eGFR. Multiple linear regression analysis was performed by using eGFR as a dependent variable to evaluate the role of the parameters in eGFR. All variables with *P* < 0.20 on univariate linear regression analyses were included in multiple linear regression analysis. Both Beta and 95% CI were estimated.

To compare the prevalence of kidney disease outcomes of different urine pH metrics, multivariable logistic regression models were constructed. Model 1 represented unadjusted odd ratios (ORs). Model 2 included demographic characteristics, such as age, sex, duration of gout, presence of tophus, and SU. In model 3, further covariates were added to model 2, including history of hypertension and diabetes. eGFR was divided into Yes or No according to whether it was > stage 1 CKD. Furthermore, we examined the effect modification for kidney disease outcomes in prespecified subgroups by SU (> or ≤ 480 μmol/L, SU level for initiation of urate-lowering therapy according to the 2016 updated EULAR evidence-based recommendations for the management of gout [28], as well as SU level cut-off point of recurrence rate of gout > 50% [29]). All statistical analysis was performed with SPSS v22.0 (IBM SPSS, Chicago, USA) software. *P* < 0.05 was considered statistically significant.

## Results

### General parameters assessed

**Fig. 1:**
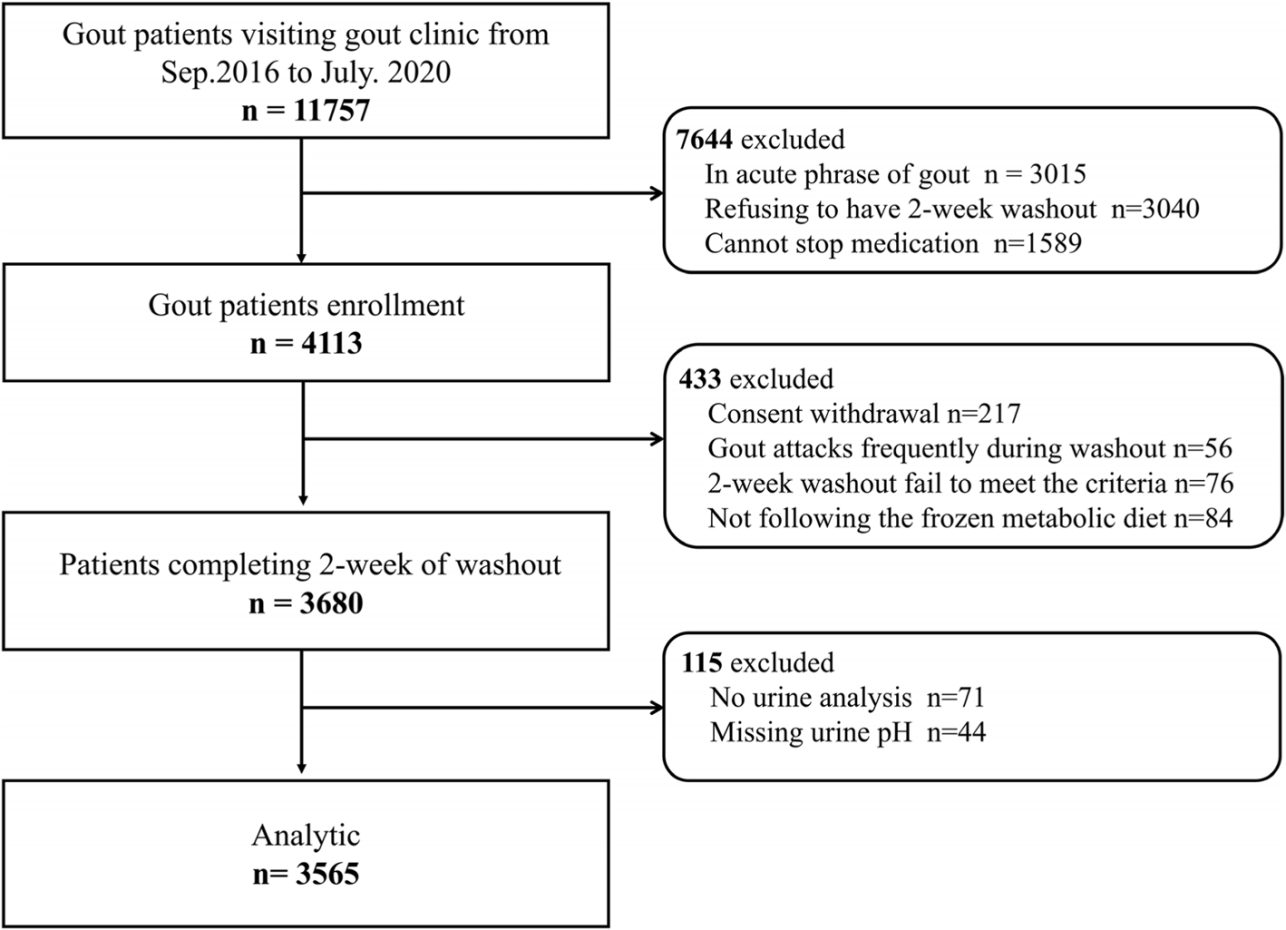
The flow chart of the cross-sectional study

In this study, of the 11,757 gout patients screened, 3565 patients were selected for analysis (Fig. 1). The main characteristics of the 3565 primary gout patients included in the study are shown in Table 1. Most were males (96.2%). The median age was 43 (IQR 33~56) years, median estimated gout duration 4 (IQR, 1.3~8.0) years, and median serum urate 517 (IQR, 453~583) μmol/L. Three were receiving regular urate-lowering therapy (ULT), even though 18.5% had a history of ULT for a generally short period. In total, 688 (19.3%) had at least one palpable tophaceous gout, 596 (16.7%) had a history of nephrolithiasis, 156 (4.4%) had type 2 diabetes, and 884 (24.8%) had hypertension. The median urine pH of all patients was 5.63 (IQR 5.37~6.09), and median eGFR was 98.32 (IQR 86.03~110.60) mL/min/1.73m^2^. Basic characteristics were compared between five groups (pH ≤ 5.0, 5.0 < pH ≤ 5.5, 5.5 < pH < 6.2, 6.2 ≤ pH ≤ 6.9, and pH > 6.9). Significant differences were found in sex, age, BMI, gout disease duration, smoking history, alcohol history, history of nephrolithiasis, systolic blood pressure (SBP), ALT, AST, TG, TC, sCr, BUN, SU, uCr, uUA, UUE, and FEUA among the groups (Table 1).

**Table 1:** Baseline characteristics of patients with different urine pH

Parameters	All patients	pH≤5.0	5.0<pH≤5.5	5.5<pH<6.2	6.2≤pH≤6.9	pH>6.9	*P* value
*n* (%)	3565	574 (16.1)	1082 (30.4)	1058 (29.7)	515 (14.5)	336 (9.4)	-
Sex							<0.001
Male, *n* (%)	3429 (96.2)	539 (93.9)	1041 (96.2)	1016 (96.0)	480 (93.2)	303 (90.2)	-
Female, *n* (%)	136 (3.8)	35 (6.1)	41 (3.8)	42 (4.0)	35 (6.8)	33 (9.8)	-
Age, years	43 (33, 56)	47 (35, 62)^**^	42 (33, 54)	42 (32, 55)	42 (33, 56)	43 (33, 58)	<0.001
BMI, kg/m^2^	26.58 (3.59)	26.55 (3.72)	26.93 (3.61)^**^	26.60 (3.59)	26.43 (3.38)	25.67 (3.46)^**^	<0.001
Duration of gout, years	4 (1.3, 8)	5 (2, 9.4)	4 (1.7, 8.0)	4 (1.7, 8.0)	3.6 (1.0, 8.0)	1.0 (1.0, 4.7)	<0.001
Alcohol history, *n* (%)	2317 (65.0)	342 (59.6)	779 (72.0)	697 (65.9)	310 (60.2)	189 (56.3)	<0.001
Smoking history, *n* (%)	1580 (44.3)	269 (46.9)	519 (48.0)	456 (43.1)	209 (40.6)	127 (37.8)	<0.01
Hypertension, *n* (%)	884 (24.8)	185 (32.2)	240 (22.2)	247 (23.3)	132 (25.6)	80 (23.8)	<0.001
Type 2 diabetes, *n* (%)	156 (4.4)	40 (7.0)	38 (3.5)	40 (3.8)	26 (5.0)	12 (3.6)	<0.05
Tophus, *n* (%)	688 (19.3)	116 (20.2)	230 (21.3)	230 (21.7)	86 (16.7)	26 (7.7)	<0.001
History of renal lithiasis							<0.001
Yes, *n* (%)	596 (16.7)	108 (18.8)	172 (15.9)	167 (15.8)	83 (16.1)	66 (19.6)	
No, *n* (%)	2193 (61.5)	286 (49.8)	763 (70.5)	754 (71.3)	293 (56.9)	97 (28.9)	
Unknown, *n* (%)	776(21.8)	180 (31.4)	147 (13.6)	137 (12.9)	139 (27.0)	173 (51.5)	
Use of medicine before enrolment							
Regular ULT, *n* (%)	3 (0.08)	0	2 (0.18)	1 (0.09)	0	0	0.641
Occasional ULT, *n* (%)	660 (18.5)	78 (13.6)	229 (21.2)	225 (21.3)	90 (17.5)	38 (11.3)	<0.001
NSAIDs/ steroids for gout flares	442 (12.4)	60 (10.5)	146 (13.5)	155 (14.7)	66 (12.8)	15 (4.5)	<0.001
SBP, mmHg	130 (120, 140)	129 (120, 140)	131 (120, 141)^**^	130 (120, 140)	128 (118, 140)	129 (119, 138)	<0.001
DBP, mmHg	82 (76, 90)	82 (77, 90)	83 (76, 90)^*^	81 (75, 90)	81 (74, 90)	82 (77, 90)	0.143
ALT, U/L	26 (18, 39)	27 (18, 41)^**^	26(19, 41)^**^	26 (18, 39)^*^	24 (18, 36)	26 (17, 38)	<0.05
AST, U/L	21 (17, 26)	22 (18, 28)^**^	21 (17, 26)	21 (17, 26)	21 (17, 25)	21 (17, 26)	<0.05
FBG, mmol/L	5.42 (5.08, 5.87)	5.46 (5.07, 5.92)	5.45 (5.08, 5.88)	5.45 (5.06, 5.88)	5.37 (5.07, 5.82)	5.33 (5.06, 5.77)	0.06
TG, mmol/L	1.82 (1.27, 2.65)	1.93 (1.32, 2.77)^**^	1.92 (1.35, 2.83)^**^	1.68 (1.19, 2.42)	1.79 (1.26, 2.47)	1.85 (1.30, 2.48)	<0.001
TC, mmol/L	4.90 (0.96)	4.93 (1.05)	4.94 (0.91)	4.85 (0.97)	4.85 (0.93)	5.01 (0.99)^*^	<0.05
HDL, mmol/L	1.07 (0.27)	1.06 (0.26)^*^	1.05 (0.27)^*^	1.06 (0.28)	1.09 (0.26)	1.13 (0.27)^**^	<0.001
LDL, mmol/L	3.25 (0.93)	3.19 (0.91)	3.31 (0.95)	3.24 (0.97)	3.21 (0.88)	3.22 (0.87)	0.089
BUN, mmol/L	4.50 (3.80, 5.40)	4.70 (3.90, 5.75)^**^	4.60 (3.80, 5.50)^**^	4.40 (3.70, 5.30)	4.40 (3.70, 5.20)	4.30 (3.63, 5.28)	<0.001
sCr, μmol/L	85.20 (18.32)	89.31 (22.35)^**^	85.63 (18.12)^**^	84.88 (16.65)^**^	81.94 (17.74)	82.85(15.71)	<0.001
SU, μmol/L	517 (453, 583)	531 (471, 600)^**^	526 (461, 597)^**^	515 (451, 583)^**^	496 (437.3, 550)	487 (417, 548)	<0.001
eGFR, mL/min/1.73m^2^	98.32 (86.03, 110.6)	93.81 (81.33, 107.9)^**^	97.70 (86.12, 109.8)^**^	98.66 (86.97, 110.8)^**^	101.8(89.02, 112.4)	99.02 (87.26, 111.8)	<0.001
uCr, μmol/L	6619 (4205, 13146)	8830 (4812, 14406)^**^	5688 (4025, 11791)	5944 (4096, 12087)	6162 (4025, 12660)	11771 (5652, 15,229)^**^	<0.001
uUA, μmol/L	1776 (1049, 3167)	2147 (1197, 3367)^**^	1484 (935, 2850)^*^	1673 (1014, 2999)	1746 (1046, 3128)	2753 (1526, 3923)^**^	<0.001
UUE, mg/d/1.73 m^2^	616.91 (445.10, 1031.52)	633.70 (441.72, 1118.87)	578.08 (424.98, 868.95)^**^	599.31 (455.90, 932.97)	639.48 (435.36, 1067.49 )	507.38 (427.51, 667.95)^**^	<0.001
FEUA, %	4.13 (3.34, 4.98)	4.04 (3.21, 5.03)^**^	3.97 ( 3.22, 4.69)^**^	4.19 (3.38, 5.08)	4.33 (3.54, 5.15)	4.24 (3.55, 5.46)	<0.001
pH	5.63 (5.37, 6.09)	5.00 (5.00, 5.00)^**^	5.50 (5.34, 5.50)^**^	6.00 (5.80, 6.00)^**^	6.50 (6.50, 6.50)	7.00 (7.00, 7.00 )	<0.001

Values for continuous variables are described as mean (SD) or median (IQR) depending on the distribution, and for categoric variables described as count (%). ANOVA and chi-squared tests were used for continuous and categorical variables. The Kruskal–Wallis test was used for variables with a skewed distribution. Comparisons were referred to group 6.2 ≤ pH ≤ 6.9. * *P* < 0.05; ** *P* < 0.01.

*BMI* body mass index, *SBP* systolic blood pressure, *DBP* diastolic blood pressure, *1 mmHg*=0.133kPa, *ALT* alanine aminotransferase, *AST* aspartate aminotransferase, *FBG* fasting blood glucose, *TG* triglycerides, *TC* total cholesterol, *HDL* high-density lipoprotein, *LDL* low-density lipoprotein, *BUN* blood urea nitrogen, *sCr* serum creatinine, *eGFR* estimate glomerular filtration rate, *SU* serum urate, *uCr* urine creatinine, *uUA* urine uric acid, *UUE* 24-h urinary urate excretion, *FEUA* fractional excretion of urate

### Urine pH is independently associated with eGFR

The results of multivariate analysis are summarized in Table 2. eGFR was associated with age (Beta, −0.90, 95% CI, −0.94 ~ -0.86; *P* < 0.001), sex (Beta, 5.07, 95% CI, 2.06 ~ 8.08; *P*=0.001), DBP (Beta, −0.09, 95% CI, −0.15 ~ -0.02; *P*=0.008), SU (Beta, −0.05, 95% CI, −0.05 ~ −0.04; *P*<0.001), hypertension (Beta, −2.32, 95% CI, −3.68 ~ −0.96; *P*=0.001), and urine pH (Beta, 1.21, 95% CI, 0.38 ~ 2.04; *P*=0.004) (Table 2).

**Table 2:** Simple and multiple linear regression of determinants of eGFR

	Univariate analysis		Multivariate analysis	
	Beta (95%CI)	*P* value	Beta (95%CI)	*P* value
Age, years	−0.87 (−0.91~−0.84)	<0.001	−0.90 (−0.94~−0.86)	<0.001
Sex (Male)	12.06 (8.53~15.58)	<0.001	5.07 (2.06~8.08)	0.001
Duration of gout, years	−0.54 (−0.66~−0.43)	<0.001	0.08 (−0.03~0.18)	0.16
tophus	−4.63 (−6.35~−2.91)	<0.001	−1.03 (−2.48~0.42)	0.17
BMI, kg/m^2^	0.30 (0.11~0.49)	0.002	0.07 (−0.09~0.23)	0.37
SBP, mmHg	−0.20 (−0.24~-0.16)	<0.001	0.04 (−0.01~0.08)	0.10
DBP, mmHg	−0.25 (−0.31~-0.19)	<0.001	−0.09 (−0.15~-0.03)	0.008
FBG, mmol/L	−2.78 (−3.47~−2.10)	<0.001	0.44 (−0.17~1.05)	0.16
TG, mmol/L	0.04 (−0.32~0.41)	0.81	-	
TC, mmol/L	−2.16 (−2.86~−1.46)	<0.001	−0.07 (−0.64~0.50)	0.81
SU, μmol/L	−0.02 (−0.02~−0.01)	<0.001	−0.05 (−0.05~−0.04)	<0.001
Urine pH	2.80 (1.77~3.84)	<0.001	1.21 (0.38~2.04)	0.004
Hypertension	−13.23 (−14.74~−11.71)	<0.001	−2.32 (−3.68~−0.96)	0.001
Diabetes	−12.51 (−15.77~−9.25)	<0.001	−2.85 (−5.74~0.04)	0.05

*BMI* body mass index, *SBP* systolic blood pressure, *DBP* diastolic blood pressure; 1 mmHg=0.133kPa, *FBG* fasting blood glucose, *TG* triglycerides, *TC* total cholesterol, *eGFR* estimate glomerular filtration rate, *SU* serum urate

### Association of urine pH subgroups with prevalence of kidney disease outcomes

The proportions of patients in each pH subgroup were 16.1% (urine pH ≤ 5.0), 30.4% (5.0 < urine pH ≤ 5.5), 29.7% (5.5 < urine pH < 6.2), 14.5% (6.2 ≤ urine pH ≤ 6.9), and 9.4% (urine pH > 6.9) (Supplementary Fig. 1A). In total, 1656 (46.5%) patients manifested an acidic urine. Our data showed that the lowest eGFR and the highest prevalence of nephrolithiasis were in patients with urine pH ≤ 5.0 (Supplementary Fig. 1B, 1C and 1D). The high prevalence of kidney cysts was observed in patients with pH ≤ 5.0 (19.5%) and pH > 6.9 (19.8%) (Supplementary Fig. 1F). Patients with 6.2 ≤ urine pH ≤ 6.9 had the highest eGFR and the lowest prevalence of urinary protein and microhematuria (Supplementary Fig. 1B, 1G and 1H).

Logistic regression analysis models also showed significantly higher risk of kidney disease outcomes in patients with urine pH ≤ 5.0 than in those with 6.2 ≤ pH ≤ 6.9. After adjustment for confounding factors, urine pH ≤ 5.0 was associated with a 1.6-fold higher risk of > stage 1 CKD (model 3, OR 1.61, 95% CI 1.15 ~ 2.25; *P* = 0.005), a 1.5-fold higher risk of nephrolithiasis (model 3, OR 1.51, 95% CI 1.07 ~ 2.14; *P* = 0.02), a 1.6-fold higher risk of kidney cyst (model 3, OR 1.69, 95% CI 1.10 ~ 2.59; *P* = 0.017), and 1.7-fold higher risk of microhematuria (model 3, OR 1.77, 95% CI, 1.29 ~ 2.44; *P* < 0.001) (Table 3). Moreover, urine pH > 6.9 was associated with a 2-fold higher risk of kidney cyst (model 3, OR 2.08, 95% CI 1.28 ~ 3.39; *P* = 0.003) and 1.8-fold higher risk of microhematuria (model 3, OR 1.79, 95% CI 1.24 ~ 2.58; *P* = 0.002) (Table 3).

**Table 3:** Association of urine pH subgroups with prevalence of kidney disease outcomes

	Model 1		Model 2		Model 3	
	OR (95% CI)	*P* value	OR (95% CI)	*P* value	OR (95% CI)	*P* value
> Stage 1 CKD						
pH ≤ 5.0	2.26 (1.76~2.91)	<0.001	1.74 (1.28~2.37)	<0.001	1.61 (1.15~2.25)	0.005
5.0 < pH≤ 5.5	1.38 (1.10~1.73)	0.006	1.31 (1.00~1.73)	0.05	1.20 (0.89~1.62)	0.22
5.5 < pH < 6.2	1.17 (0.93~1.47)	0.18	1.19 (0.90~1.57)	0.23	1.17 (0.87~1.59)	0.30
6.2 ≤ pH ≤ 6.9	Reference	-	Reference	-	Reference	-
pH > 6.9	1.33 (0.99~1.79)	0.06	1.31 (0.90~1.90)	0.16	1.12 (0.75~1.69)	0.58
Nephrolithiasis						
pH ≤ 5.0	2.12 (1.56~2.87)	<0.001	1.71 (1.25~2.34)	0.001	1.51 (1.07~2.14)	0.02
5.0 < pH≤ 5.5	0.86 (0.64~1.16)	0.32	0.76 (0.56~1.04)	0.088	0.55 (0.39~0.78)	0.001
5.5 < pH < 6.2	0.98 (0.73~1.31)	0.87	0.90 (0.66~1.22)	0.486	0.80 (0.57~1.12)	0.20
6.2 ≤ pH ≤ 6.9	Reference	-	Reference	-	Reference	-
pH > 6.9	1.19 (0.82~1.73)	0.365	1.08 (0.73~1.60)	0.706	1.14 (0.74~1.76)	0.55
Kidney cyst						
pH ≤ 5.0	2.24 (1.56~3.21)	<0.001	1.69 (1.14~2.51)	0.009	1.69 (1.10~2.59)	0.02
5.0 < pH≤ 5.5	1.39 (0.99~1.97)	0.06	1.39 (0.96~2.02)	0.08	1.26 (0.84~1.90)	0.26
5.5 < pH < 6.2	1.21 (0.85~1.71)	0.29	1.31 (0.90~1.91)	0.16	1.31 (0.87~1.96)	0.20
6.2 ≤ pH ≤ 6.9	Reference	-	Reference	-	Reference	-
pH > 6.9	2.28 (1.53~3.41)	<0.001	2.12 (1.37~3.30)	0.001	2.08 (1.28~3.39)	0.003
Microhematuria						
pH ≤ 5.0	1.94 (1.48~2.52)	<0.001	1.94 (1.47~2.57)	<0.001	1.77 (1.29~2.44)	<0.001
5.0 < pH≤ 5.5	1.37 (1.07~1.77)	0.013	1.44 (1.11~1.87)	0.007	1.19 (0.87~1.61)	0.28
5.5 < pH < 6.2	1.31 (1.02~1.69)	0.035	1.41 (1.08~1.83)	0.011	1.35 (1.00~1.83)	0.053
6.2 ≤ pH ≤ 6.9	Reference	-	Reference	-	Reference	-
pH > 6.9	1.50 (1.11~2.03)	0.009	1.39 (1.01~1.92)	0.042	1.79 (1.24~2.58)	0.002
Proteinuria						
pH ≤ 5.0	3.44 (1.71~6.92)	<0.001	2.41 (1.18~4.93)	0.016	1.86 (0.85~4.07)	0.12
5.0 < pH≤ 5.5	3.79 (1.92~7.46)	<0.001	3.42 (1.72~6.81)	<0.001	2.83 (1.33~6.01)	0.007
5.5 < pH < 6.2	3.17 (1.60~6.31)	<0.001	3.02 (1.50~6.06)	0.002	2.19 (1.02~4.72)	0.044
6.2 ≤ pH ≤ 6.9	Reference	-	Reference	-	Reference	-
pH > 6.9	1.82 (0.80~4.14)	0.16	1.32 (0.56~3.14)	0.53	0.88(0.32~2.39)	0.80

Model 1 represents unadjusted ORs. Model 2 included demographic characteristics, such as age, sex, duration of gout, presence of tophi and SU. In model 3, further covariates were added to model 2, including history of hypertension and diabetes

### SU and urine pH subgroup analysis

**Fig. 2:**
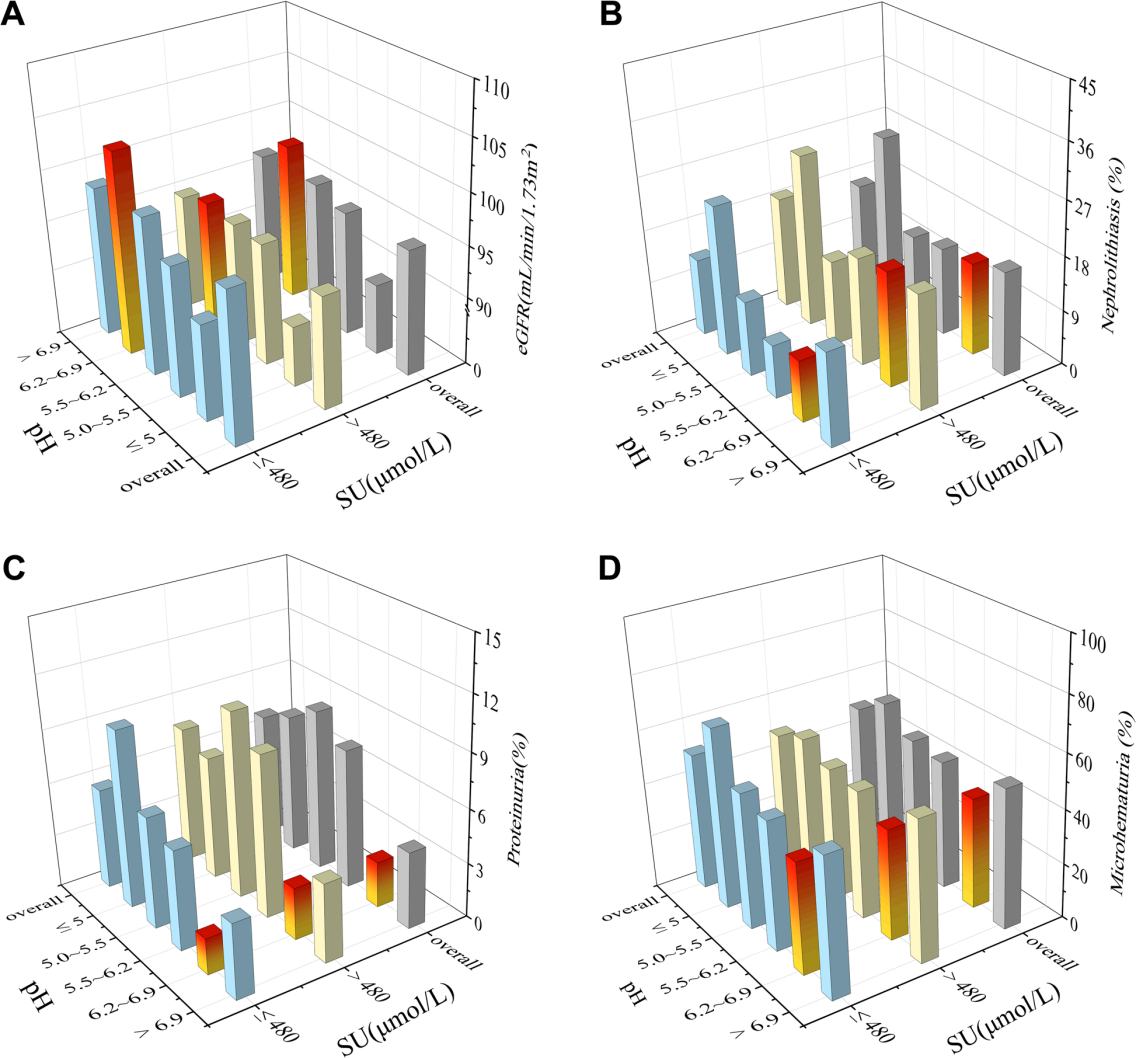
The eGFR and prevalence of kidney disease outcomes in subgroups based on SU and urine pH values. **A** eGFR. **B** Nephrolithiasis. **C** Proteinuria. **D** Microhematuria. eGFR estimated glomerular filtration rate

Given the mutual influence of urate, urine pH, and prevalence of kidney disease outcomes in patients with gout, we subgrouped the patients according to their SU and urine pH values. Patients with SU ≤ 480 μmol/L and 6.2 ≤ urine pH ≤ 6.9 had the highest eGFR and lowest proportion of patients with proteinuria (2.0%) (Fig. 2A, C). We also found that the prevalence of nephrolithiasis in patients with SU ≤ 480 μmol/L to be lower than patients with SU > 480 μmol/L (13.0% *vs.* 19.0%, *P* <0.01; Fig. 2B). The same trend was shown in patients with microhematuria. Of note, the subgroups of both SU ≤ 480 μmol/L and SU > 480 μmol/L had significantly lower and similar prevalence of microhematuria when 6.2 ≤ urine pH ≤ 6.9 (40.9% *vs.* 40.9%; Fig. 2D).

We examined the effect modification by SU. The significant association of urine pH subgroups with the prevalence of kidney disease outcomes, including nephrolithiasis, microhematuria, and proteinuria was consistent across subgroup by SU. However, there was a significant interaction between SU and urine pH (*P* value for interaction 0.027) for incident > stage 1 CKD (Table 4). In the subgroup with SU < 480 μmol/L, participants with 5.0 < urine pH ≤ 5.5 had a 2.2-fold higher risk of > stage 1 CKD than those with 6.2 ≤ urine pH ≤ 6.9 (OR 2.20, 95% CI 1.48 ~ 3.26; *P*<0.001). Nevertheless, in the subgroup with SU > 480 μmol/L, participants with 5.0 < urine pH≤ 5.5 had no higher risk of > stage 1 CKD than those with 6.2 ≤ pH ≤ 6.9 (*P* = 0.773).

**Table 4:** Interaction analysis of eGFR and prevalence of kidney adverse events in subgroups based on SU and urine pH values

Kidney disease outcomes	pH ≤ 5.0	5.0 < pH≤ 5.5	5.5 < pH < 6.2	6.2 ≤ pH ≤ 6.9	pH > 6.9	*P* for interaction
1 stage CKD						0.027
SU>480	1.88 (1.38~2.55)	1.04 (0.79~1.38)	1.04 (0.78~1.38)	reference	1.20 (0.82~1.77)	
SU ≤ 480	2.90 (1.85~4.56)	2.20 (1.48~3.26)	1.375 (0.92~2.05)	reference	1.70 (1.06~2.72)	
Nephrolithiasis						0.08
SU>480	1.73 (1.20~2.45)	0.70 (0.49~1.01)	0.93 (0.66~1.33)	reference	0.99 (0.61~1.60)	
SU ≤ 480	2.98 (1.68~5.28)	1.27 (0.73~2.20)	0.860 (0.48~1.53)	reference	1.60 (0.85~3.02)	
Microhematuria						0.173
SU>480	1.66 (1.19~2.30)	1.37 (1.00~1.89)	1.30 (0.94~1.80)	reference	1.57 (1.04~2.36)	
SU ≤ 480	2.94 (1.84~4.72)	1.47 (0.97~2.22)	1.32 (0.88~1.99)	reference	1.50 (0.95~2.39)	
Proteinuria						0.234
SU>480	2.49 (1.06~5.82)	3.91 (1.74~8.78)	3.34 (1.47~7.59)	reference	1.53 (0.53~4.44)	
SU ≤ 480	5.40 (1.53~19.08)	3.25 (0.93~11.43)	2.88 (0.82~10.20)	reference	2.12 (0.52~8.62)	

## Discussion

Though gout patients have decreased urine pH overall, neither the specific distribution of the urine pH nor the association of low pH to kidney disease in gout are known. Our study was quite large and was rigorous in medication washout, use of a standardized diet, and exclusion of > stage 2 CKD, in order to excluding the confounding factors such as diet, drugs, and severe chronic renal function that impact urine pH. We also included renal ultrasound examination. We observed that nearly half of the gout patients had acidic urine, with 16.10% of the patients having urine pH less than 5.0. The proportion of patients with urine pH between 6.2 and 6.9 was 14.45%. Renal damage in gout patients was also closely related to urine pH [30]. Our results showed that gout patients with acidic urine had the significantly decreased eGFR but increased proteinuria, microhematuria, renal cyst, and nephrolithiasis.

This study found that patients with decreased eGFR had low urine pH, consistent with previous reports [31]. When urine pH is altered, renal clearance of creatinine may change dramatically. Previous studies also have shown that low urine pH was a risk factor for CKD independent of urate [32]. The trend in higher net endogenous acid production is associated with a faster rate of decline in eGFR among hypertensive CKD [33]. In CKD, as overall acid excretion is impaired, acid excretion per nephron is increased to compensate for the loss of nephrons, which in turn may further promote tubulointerstitial damage and contribute to the progression of kidney disease. When urine pH is elevated via the supplementation of sodium bicarbonate, the progression of CKD is significantly slowed down [34]. These reports strongly suggest that a lower urine pH is closely linked to the advancement of kidney damage.

Our data showed that patients with urine pH < 5.0 was associated with significantly increased proteinuria, microhematuria, renal cyst, and nephrolithiasis. More than 50% of stones in gout patients are uric acid stones in which the driving biochemical abnormalities first include low urine pH [35, 36], but also high urine urate concentration and low volume [37]. The prevalence of urate nephrolithiasis increased significantly when urine pH was below 5.5 in this study [38]. At pH 5.5, half of 600 mg of urate in 1L of urine is predicted to be insoluble [38], likely contributing to the significantly increased prevalence of both solitary or multiple kidney stones in gout patients with low pH in this study. In addition, low pH may reduce the reabsorption of phosphorus by inhibiting NaPi IIA, resulting in phosphaturia and kidney stones [39, 40]. Genetic and other factors, such as high BMI, transport protein channels and purine metabolism could have influenced kidney stone prevalence [41, 42].

Microhematuria and proteinuria are important indicators of kidney damage [43]. Our data showed the prevalence of microhematuria in gout patients was 49.5%, obviously higher than that in healthy population (2.4~31.1%) [23]. It was well known that microhematuria can be asymptomatic; however, a study to evaluate the risk of end-stage kidney disease (ESKD) in adolescents and young adults with persistent asymptomatic isolated microhematuria showed that microhematuria was associated with increased risk of treated ESKD [44]. Hyperuricosuria is another reason of microhematuria, and it has been reported that allopurinol plus forced water intake can decrease the microhematuria [45]. Determining the etiology of trace hematuria is beyond the scope of the study, but one possibility is that uric acid microcrystals in acidic urine may physically damage the epithelium of the urinary collection system and lead to hematuria.

Although crystals in urine have not been detected in our study, low urinary pH has been reported to be the most important factor leading to uric acid crystallization [35]. Protonated urate, the predominant form of uric acid in urine at 37°C when the urine pH is close to the first dissociation constant (pKa) of 5.5 is substantially less soluble than urate salts, favoring its precipitation [46]. Crystal formation or crystallization begins with nucleation, in which molecules and/or ions gather and self-organize in space with defined orientation, distance, and symmetry [47]. When urine pH is less than 5.5, uric acid is supersaturated in urine resulting in uric acid crystallization, which is characteristic of uric acid. Normal uric acid excretion is less than 750 mg/day for women and 800 mg/day for men, but in acidic urine, uric acid can crystallize at levels as low as 200 mg/day [48]. Recent findings suggested that CKD may not progress unless uric acid crystallizes in the kidney, a pro-inflammatory and cytotoxic event [49]. Therefore, correcting too low urinary pH or further reducing the quantity of uric acid in urine will be an effective treatment to avoid uric acid crystallization and even kidney stone formation [11]. However, excessive alkalinization of urine can result in a urine pH of over 7 which increases the risk of forming calcium phosphate calculi [48].

We observed a linear relationship between renal uric acid excretion and urine pH. A decrease in urine pH occurs because of an increased supply of hydrogen ions to the urine or decreased elimination of urinary H^+^. The change of urine H^+^ concentration in gout patients caused by several factors. Higher uric acid is more likely to form uric acid crystals, which can cause tubular damage. In addition, elevated uric acid can directly stimulate the production of reactive oxygen species, chemotactic cytokines, and inflammation, leading to the renal tubular injury. The capacity of producing NH4^+^ and excretion of NH3 was decreased in abnormal renal tubules. The higher serum uric acid level of gout patients, the components of metabolic syndrome complicated with diabetes, hyperlipidemia, and other components increased significantly. Insulin resistance is the common pathophysiological basis of metabolic syndrome. Insulin not only promotes the secretion of H^+^ by increasing Na^+^-H^+^ exchange, but also reduces the synthesis and excretion of NH3 and affects urine pH. In turn, low urinary pH also predicts higher serum uric acid levels and exacerbates kidney damage. Our data showed the patients with acidic pH had lower excretion of urinary urate (Table 1), which may be caused by increased crystallization and deposition of urate. When urine pH increased from 5.9 to 6.5, renal uric acid excretion was enhanced correspondingly from 302 to 413 mg/day [50]. In addition, increased urate reabsorption may be another cause of reduced urate excretion. Although pH did not affect transport of urate for URAT1 [51], it had been reported that URAT1 expression increased in renal tubules when insulin resistance, increasing the reabsorption of urate and then leading to the lower excretion of urinary urate and higher serum urate levels [52].

In those patients with serum urate levels both above and below the level of 480μmol/L, there was lower eGFR and the higher incidence of renal-related disease outcomes in patients with pH < 5. Because patients with moderate uric acid excretion but fixed low urine pH can form uric acid stones, it appears that reducing uric acid excretion with xanthine oxidase/dehydrogenase inhibitors (allopurinol and febuxostat) without increasing urine pH will ultimately not be as successful as alkalinization [11]. Our data showed that patients with 6.2 ≤ urine pH ≤ 6.9 and SU ≤ 480 μmol/L had the highest eGFR with the lowest prevalence in nephrolithiasis, urinary protein, and microhematuria.

Limitations of this study included that the cross-sectional, non-interventional research design, which was undertaken for hypothesis-testing and to provide a potential foundation for interventional trial urine alkalinization in gout. We excluded patients who did not meet the criteria for inclusion in this study, leaving it to be seen whether the results would apply to other geographic regions, ethnicities, or environments. Our work analyzed the prevalence of nephrolithiasis, but did not assess composition of renal calculi. We did not study a control group of asymptomatic hyperuricemia or focus separately on those with frequently gout-associated comorbidities, such as hypertension, obesity, metabolic syndrome, and type 2 diabetes. Only selected blood and urine indicators were analyzed in the current study, whereas serum and urine bicarbonate and urine ammonia may have been informative. Additionally, urine crystals and markers of renal tubular injury were not measured in this study.

## Conclusion

In conclusion, the acidic urine pH of ~50% Chinese gout patients or above-average urine pH (pH > 6.9) were associated with increased prevalence of proteinuria, microhematuria, renal cyst, and nephrolithiasis. Urate-lowering treatment to target SU is a lifelong strategy for gout patients [20]. However, the role of therapeutic urine alkalization remains unclear, due to lack of evidence from prospective, controlled clinical trials, as well as lack of use of optimal urine pH monitoring approache s[20, 53]. Our results support further, prospective clinical investigations into the potential benefits of urine alkalization in gout patients with acid urine and hyperuricemia.

## Supplementary Information

**Additional file 1:** **Supplementary Table 1.** The metabolic diet. **Supplementary Figure 1.** Distribution, kidney function and prevalence of kidney disease outcomes in patients with different urine pH values. (A) the distribution; (B) eGFR; (C) kidney stone; (D) solitary kidney stone; (E) multiple kidney stones; (F) kidney cyst; (G) urine protein; (H) hematuria. Chi-squared tests were used for categorical variables. The chi-square test of 2*2 used for pairwise comparison between groups and significance was corrected by Bonferroni. *P* < 0.005 was considered statistically significant. *P.

## Data Availability

The datasets used and analyzed during the current study are available from the corresponding author on reasonable request.

## Abbreviations

ALT: Alanine aminotransferase
AST: Aspartate aminotransferase
sCr: Serum creatinine
FBG: Fasting blood glucose
TC: Cholesterol
TG: Triglyceride
SU: Serum urate
CKD: Chronic kidney disease
ACR: American College of Rheumatology
HDL: High-density lipoprotein
LDL: Low-density lipoprotein
uua: Urine uric acid
uCr: Urine creatinine
GFR: Glomerular filtration rate
FEUA: Fractional excretion of urate
UUE: Urinary urate excretion
SD: Standard deviation
IQR: Interquartile range
BMI: Body mass index
OR: Odd ratio
CI: Confidence interval
URAT1: Urate transporter 1
ESKD: End-stage kidney disease
