# Supplementary material for: Association of acidic urine pH with impaired renal function in primary gout patients: a Chinese population-based cross-sectional study
Source: Arthritis Res Ther. 2022 Jan 25;24:32. doi: 10.1186/s13075-022-02725-w (PMC8787907; doi:10.1186/s13075-022-02725-w)

Supplementary Table 1. The metabolic diet.

Supplementary Figure 1. Distribution, kidney function and prevalence of kidney disease outcomes in patients with different urine pH values. **(A)** the distribution; **(B)** eGFR; **(C)** kidney stone; **(D)** solitary kidney stone; **(E)** multiple kidney stones; **(F)** kidney cyst; **(G)** urine protein; **(H)** hematuria. Chi-squared tests were used for categorical variables. The chi-square test of 2*2 used for pairwise comparison between groups and significance was corrected by Bonferroni. $P < 0.005$ was considered statistically significant. *P <0.005, **P <0.001. eGFR: estimated glomerular filtration rate.

Supplementary Table 1. The metabolic diet.

Breakfast	Lunch	Dinner	Fruits
Flour 75 g	Rice 125 g or flour 125 g	Flour 75 g	150 g
Vegetables 50 g	Two kinds of vegetables, 300 g in total	Vegetables 200 g	
One egg or low-fat milk 250 mL	Pork, beef or chicken 30 g	Two eggs, or one egg and low-fat milk 250 mL	
	Blend oil 15 g	Blend oil 10 g	

Supplementary Figure 1. Distribution, kidney function and prevalence of kidney disease outcomes in patients with different urine pH values.

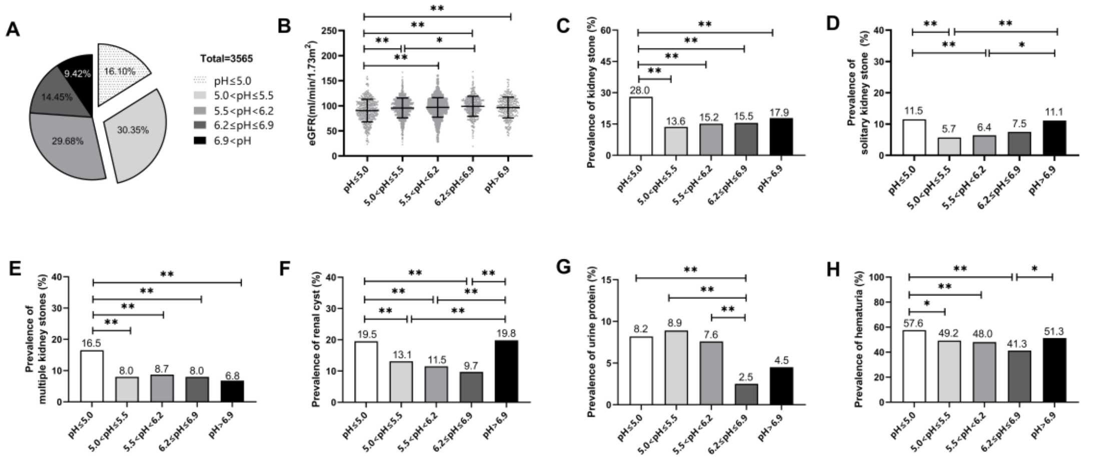

Supplement: Supplementary file 1 — Additional file 1: Supplementary Table 1. The metabolic diet. Supplementary Figure 1. Distribution, kidney function and prevalence of kidney disease outcomes in patients with different urine pH values. (A) the distribution; (B) eGFR; (C) kidney stone; (D) solitary kidney stone; (E) multiple kidney stones; (F) kidney cyst; (G) urine protein; (H) hematuria. Chi-squared tests were used for categorical variables. The chi-square test of 2*2 used for pairwise comparison between groups and significance was corrected by Bonferroni. P < 0.005 was considered statistically significant. *P. [file 13075_2022_2725_MOESM1_ESM.pdf]
